# Influence of β-lactam pharmacodynamics on the systems microbiology of gram-positive and gram-negative polymicrobial communities

**DOI:** 10.3389/fphar.2024.1339858

**Published:** 2024-06-04

**Authors:** Nicholas M. Smith, Harpreet Kaur, Ravneet Kaur, Trisha Minoza, Michael Kent, Ayeh Barekat, Justin R. Lenhard

**Affiliations:** ^1^ School of Pharmacy and Pharmaceutical Sciences, University at Buffalo, Buffalo, NY, United States; ^2^ California Northstate University College of Pharmacy, Elk Grove, CA, United States

**Keywords:** polymicrobial, pharmacodynamic, beta-lactam, mechanism-based modeling, *Staphylococcus aureus*, *Escherichia coli*, *Enterococcus faecalis*, systems microbiology

## Abstract

**Objectives:**

We sought to evaluate the pharmacodynamics of β-lactam antibacterials against polymicrobial communities of clinically relevant gram-positive and gram-negative pathogens.

**Methods:**

Two *Enterococcus faecalis* isolates, two *Staphylococcus aureus* isolates, and three *Escherichia coli* isolates with varying β-lactamase production were evaluated in static time-killing experiments. Each gram-positive isolate was exposed to a concentration array of ampicillin (*E. faecalis*) or cefazolin (*S. aureus*) alone and during co-culture with an *E. coli* isolate that was β-lactamase-deficient, produced TEM-1, or produced KPC-3/TEM-1B. The results of the time-killing experiments were summarized using an integrated pharmacokinetic/pharmacodynamics analysis as well as mathematical modelling to fully characterize the antibacterial pharmacodynamics.

**Results:**

In the integrated analysis, the maximum killing of ampicillin (E_max_) against both *E. faecalis* isolates was ≥ 4.11 during monoculture experiments or co-culture with β-lactamase-deficient *E. coli*, whereas the E_max_ was reduced to ≤ 1.54 during co-culture with β-lactamase-producing *E. coli*. In comparison to monoculture experiments, culturing *S. aureus* with KPC-producing *E. coli* resulted in reductions of the cefazolin E_max_ from 3.25 and 3.71 down to 2.02 and 2.98, respectively. Two mathematical models were created to describe the interactions between *E. coli* and either *E. faecalis* or *S. aureus*. When in co-culture with *E. coli*, *S. aureus* experienced a reduction in its cefazolin K_max_ by 24.8% (23.1%RSE). Similarly, β-lactamase-producing *E. coli* preferentially protected the ampicillin-resistant *E. faecalis* subpopulation, reducing K_max,r_ by 90.1% (14%RSE).

**Discussion:**

β-lactamase-producing *E. coli* were capable of protecting *S. aureus* and *E. faecalis* from exposure to β-lactam antibacterials.

## Introduction

Combating the proliferation of antimicrobial resistance is an international goal that requires the cooperation of the global healthcare system for success (McEwen and Collignon, 2018; Septimus, 2018; Christaki et al., 2020; Morrison and Zembower, 2020). One of the main culprits of the spread of drug-resistant bacteria is the inappropriate use of antibacterial drugs. In recognition of the importance of judicious antibacterial use, many institutions have developed antimicrobial stewardship programs that focus on ensuring that patients receive appropriate anti-infective drug regimens with optimal doses, frequencies of administration, routes of administration, and durations of therapy (Marcelin et al., 2020; Pierce et al., 2020; Lanckohr and Bracht, 2022). Looking to the future, many experts are calling for individualized antimicrobial regimens that are tailored specifically to the patient and infective pathogens (Moser et al., 2019; Bulman et al., 2022; Ko and Tsalik, 2022); however, such a paradigm will not be possible unless the medical community gains a firm understanding of how antibacterial selection and dosing are impacted by the presence of multiple pathogens at the same site of infection.

At the moment, there is a critical lack of guidance for clinicians regarding how anti-infective regimens should be modified to target a specific polymicrobial community. For example, the Infectious Diseases Society of America guidelines on skin and soft tissue infections, community-acquired pneumonia, and hospital-acquired pneumonia do not provide recommendations for antibacterial selection or dosing based on the results of polymicrobial cultures (Stevens et al., 2014; Kalil et al., 2016; Metlay et al., 2019). Although investigators have started to evaluate the pharmacokinetics and pharmacodynamics (PK/PD) of antibacterials used against multiple pathogenic organisms, the focus in the literature has largely centered on the interactions of *Staphylococcus aureus* and *Pseudomonas aeruginosa* (DeLeon et al., 2014; Hoffman et al., 2006; Orazi and O'Toole, 2017; Orazi et al., 2019; Orazi et al., 2020; Lebrun et al., 1978; Michelsen et al., 2014; Dehbashi et al., 2021; Briaud et al., 2019; Tognon et al., 2017; Beaudoin et al., 2017; Cendra et al., 2019; Radlinski et al., 2017; Lenhard et al., 2019).

Enterobacterales, an order of gram-negative enteric bacteria, are becoming increasingly notorious for the spread of drug-resistant strains (Jean et al., 2022; Lepe and Martínez-Martínez, 2022). One of the most notorious resistance mechanisms utilized by pathogenic Enterobacterales is the production of β-lactamase enzymes that inactivate many of the most clinically relevant antibacterial agents (Rabaan et al., 2022; Caliskan-Aydogan and Alocilja, 2023). In the United States, *Klebsiella pneumoniae* carbapenemase (KPC) enzymes have become the most commonly encountered carbapenemase enzyme among carbapenem-resistant Enterobacterales (CRE), and the production of the enzyme has plagued many other countries as well (Hansen, 2021). The spread of CRE was identified as an “urgent” threat to public health by the Centers for Disease Control and Prevention and a “critical” priority for drug development by the World Health Organization (WHO, 2017; Centers for Disease Control, 2023).

Not only are Enterobacterales consistently encountered during polymicrobial intra-abdominal infections (Labricciosa et al., 2018; Cornely et al., 2020), but the organisms have been co-cultured along with gram-positive pathogens such as *S. aureus* and *Enterococcus faecalis* from skin and soft tissue infections (Shettigar et al., 2016), urinary tract infections (Siegman-Igra et al., 1994; Macleod and Stickler, 2007), pneumonia (Combes et al., 2002; Ferrer et al., 2015; Patil and Patil, 2017), and bacteremia as well (Pavlaki et al., 2013; Pammi et al., 2014; Yo et al., 2019). A prior investigation confirmed that carbapenemase-producing *Acinetobacter baumannii* are capable of sheltering adjacent gram-positive organisms from β-lactam exposure, but the ability of KPC-producing Enterobacterales to protect neighboring pathogens has not been defined (Liao et al., 2014). Given the spread of CRE, the PK/PD of β-lactam antibacterials against polymicrobial infections composed of Enterobacterales and gram-positive pathogens are needed to achieve truly individualized anti-infective regimens in the future.

Models of antibiotic pharmacodynamics often implement a subpopulation model structure to describe the bacterial cell population, which includes descriptions of a ‘susceptible’ subpopulation that constitutes the majority of cells (often >99%) and a ‘resistant’ subpopulation that constitutes the minority of cells (Jacobs et al., 2016; Minichmayr et al., 2022). Ultimately, these are data-driven strategies for characterizing antibiotic effects and rely on stepwise model building. Typically, the number of subpopulations are selected empirically based on likelihood ratio testing of the model objective function, however most models in the literature only require two to three subpopulations (Bulitta et al., 2015; Landersdorfer et al., 2018; Mi et al., 2022; Minichmayr et al., 2022; Smith et al., 2022). When modeling polymicrobial infections, each bacterial specie of interest would be modeled in a similar manner. An alternative model design can be implemented that models the full distribution of susceptibility among the bacteria but can be computationally expensive (Krzyzanski and Rao, 2017). Additionally, subpopulation models can be leveraged to facilitate tracking of resistance over time if the model is fit using both total bacterial counts along with population analysis profiles, but this process is labor-intensive (Landersdorfer et al., 2013).

Here, we report the results of a PK/PD analysis of β-lactam antibacterials against mixed cultures of *Escherichia coli* and either *S. aureus* or *E. faecalis*. Duos consisting of an *E. coli* isolate and a gram-positive organism were investigated in 24-h time-killing experiments to define the time course of bacterial killing in polymicrobial conditions. To determine the relevance of β-lactamase production, several *E. coli* isolates with different β-lactamase statuses were included in the investigation. Finally, a comprehensive analysis of the data was performed using several PK/PD and pharmacometric approaches.

## Results

### Time kill studies

**FIGURE 1:**
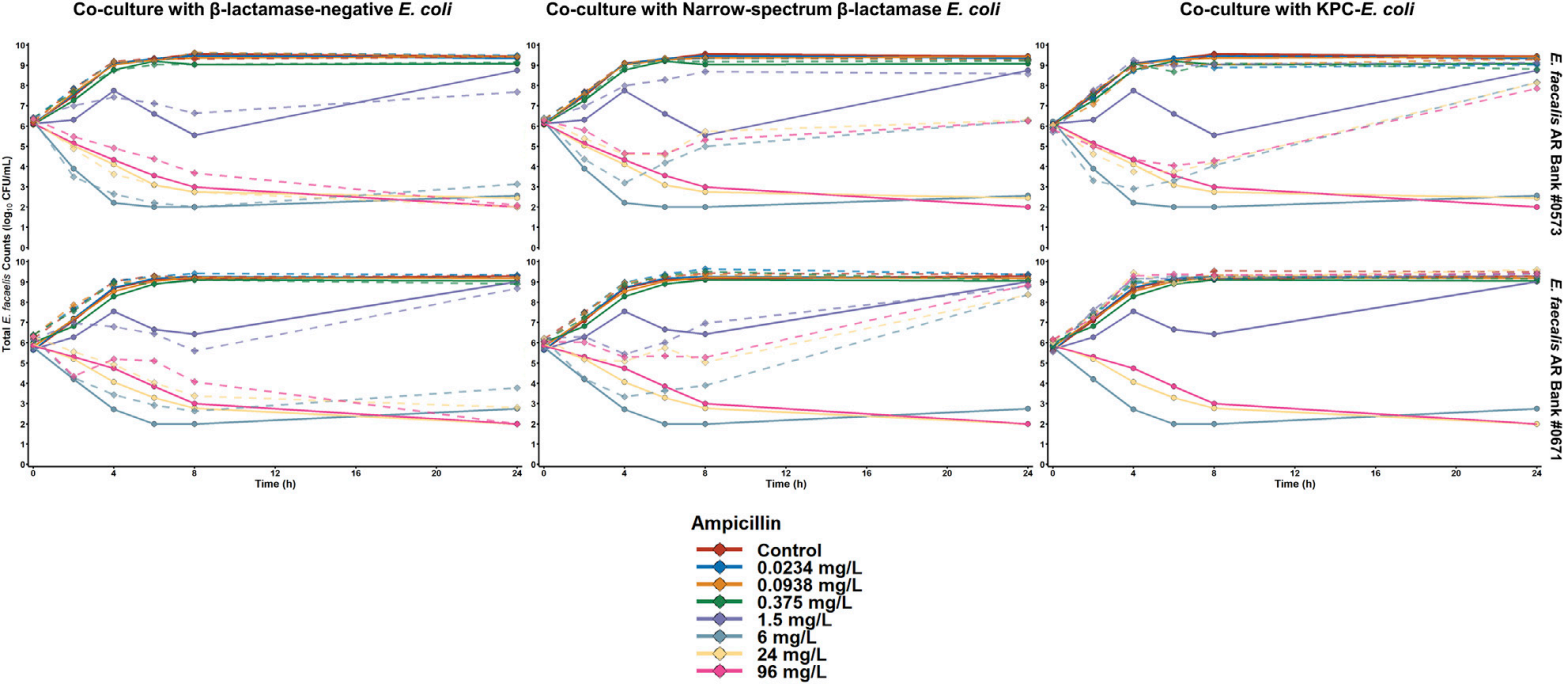
Time-killing plots depicting the activity of ampicillin against *E. faecalis* cultured alone or with *E. coli* that were either β-lactamase-deficient, produced a narrow spectrum TEM-1 enzyme, or produced TEM-1B and KPC-3. The quantity of *E. faecalis* is depicted for both monoculture (solid lines) and co-culture (dashed line) experiments.

The results of the ampicillin time-killing experiments against the *E. faecalis* isolates are summarized in Figure 1. When both *E. faecalis* isolates were cultured alone, ampicillin concentrations ≥ 6 mg/L achieved ≥ 3 log_10_ CFU/mL reductions by 24 h against both organisms. Similarly, ampicillin concentrations ≥ 6 mg/L achieved ≥ 2.5 log_10_ CFU/mL reductions by 24 h when both *E. faecalis* isolates were cultured with *E. coli* that do not produce a β-lactamase enzyme. In contrast, ampicillin concentrations up to 96 mg/L resulted in > 2 log_10_CFU/mL of *E. faecalis* growth above the starting inocula during co-culture with either of the β-lactamase-producing *E. coli* isolates. Despite having similar performance against both *E. faecalis* isolates in monoculture experiments, ampicillin was unable to achieve any killing against *E. faecalis* AR Bank # 0671 during co-culture with the KPC-producing *E. coli*, whereas ampicillin concentrations ≥ 6 mg/L reduced *E. faecalis* AR Bank #0573 counts by ≥ 1.8 log_10_ CFU/mL in the first 8 h before regrowth occurred by 24 h.

**FIGURE 2:**
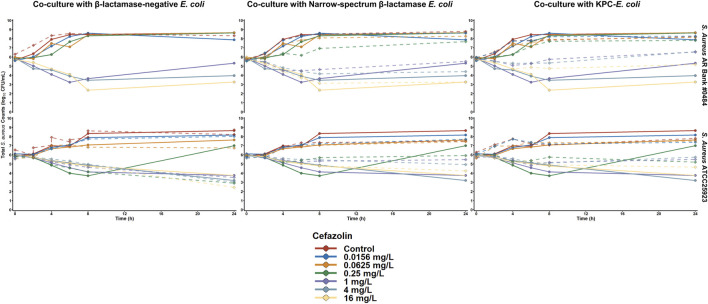
Time-killing plots depicting the activity of cefazolin against *S. aureus* cultured alone or with *E. coli* that were either β-lactamase-deficient, produced a narrow spectrum TEM-1 enzyme, or produced TEM-1B and KPC-3. The quantity of *S. aureus* is depicted for both monoculture (solid lines) and co-culture (dashed line) experiments.

The activity of cefazolin against both *S. aureus* isolates is depicted in Figure 2. When *S. aureus* ATCC 25923 was exposed to cefazolin alone or during co-culture with β-lactamase-deficient *E. coli*, cefazolin concentrations ≥ 0.25 mg/L achieved > 2 log_10_CFU/mL reductions by 24 h. In contrast, the maximum reductions of cefazolin concentrations between 0.25 and 4 mg/L were < 1 log_10_ CFU/mL during co-culture with either β-lactamase-producing *E. coli* isolate, and 16 mg/L achieved maximum reductions of 1.7 and 1.4 log_10_CFU/mL during c-culture with *E. coli* that produce TEM-1 and KPC-3/TEM-1B, respectively. Against *S. aureus* AR Bank # 0484 grown alone or cultured with *E. coli* with no β-lactamase production, cefazolin concentrations ≥ 1 mg/L achieved > 2.4 log_10_CFU/mL reductions by 24 h. During co-culture with TEM-1-producing *E. coli*, cefazolin concentrations of 1, 4, and 16 mg/L achieved maximum reductions of 1.2, 1.5, and 2.9 log_10_CFU/mL, respectively, whereas co-culture with KPC-3/TEM-1B-producing *E. coli* resulted in maximum reductions of 0.7, 0.6, and 1.1 log_10_CFU/mL, respectively.

The activity of ampicillin was evaluated against the β-lactamase-deficient *E. coli* (*E. coli* AR Bank #0017) alone and during co-culture with each of the four gram-positive isolates (Supplementary Figure S1, 2). When the *E. coli* was alone, cultured with either *E. faecalis* isolate, or cultured with the β-lactamase-deficient *S. aureus* (ATCC 25923), 96 mg/L of ampicillin reduced the *E. coli* counts below the limit of detection by 24 h. In contrast, when the *E. coli* was cultured with the penicillin-resistant *S. aureus* AR Bank # 0484, a maximum reduction of 2.2 log_10_ CFU/mL of *E. coli* was achieved at 6 h followed by regrowth to 2.6 log_10_ CFU/mL above the starting inoculum by 24 h. When the activity of cefazolin was evaluated against *E. coli* alone or cultured with each *S. aureus* isolate, 16 mg/L of cefazolin achieved a > 4.6 log_10_ CFU/mL reduction against the *E. coli* regardless of the presence of *S. aureus* (Supplementary Figure S3).

### Empiric pharmacokinetic/pharmacodynamic analysis

**FIGURE 3:**
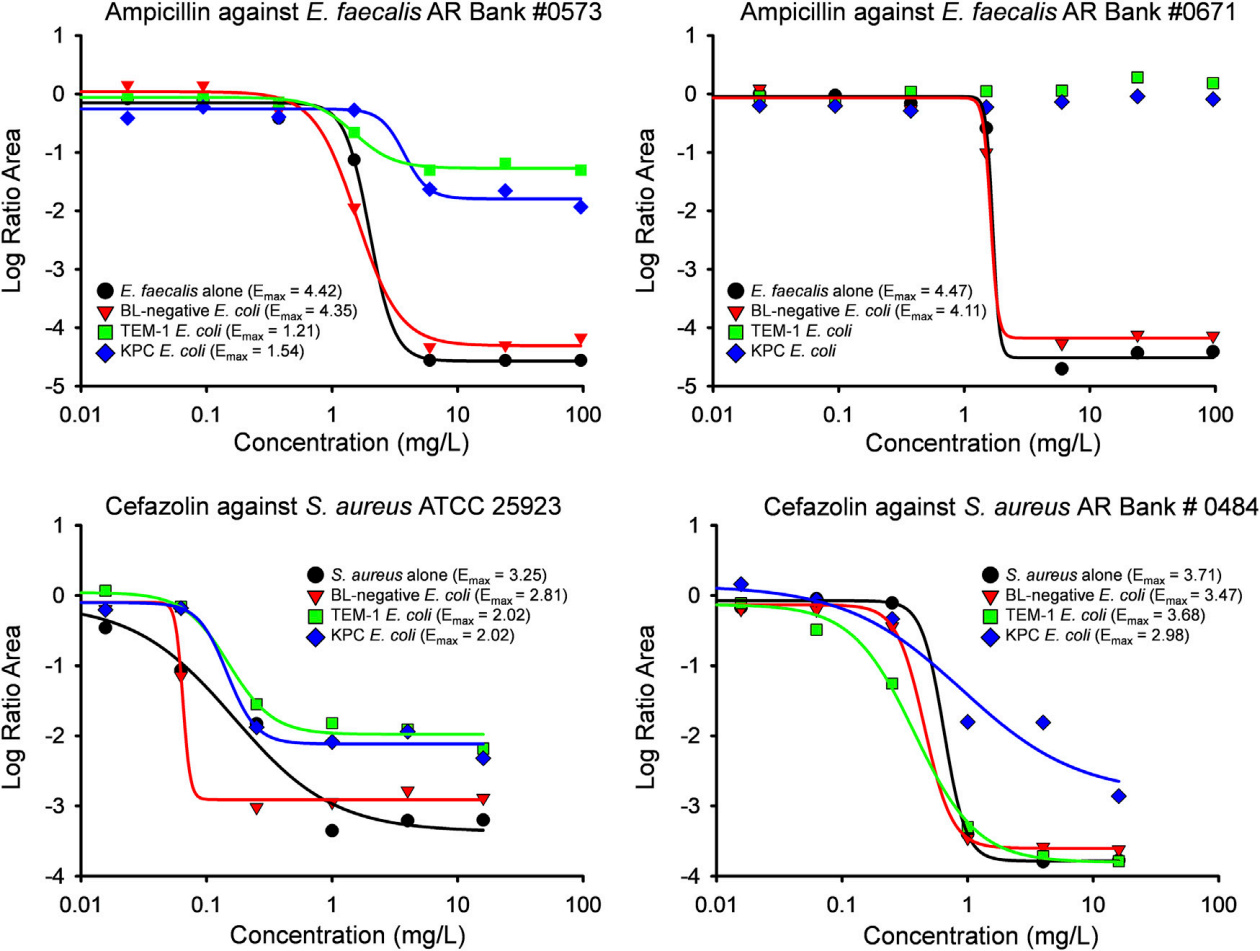
Results of an integrated PK/PD analysis are shown for both *E. faecalis* (top) and *S. aureus* (bottom) isolates investigated in the study. The Log Ratio Areas obtained from time-killing experiments were described by a mathematical Hill-type model in which E_max_ represents maximum antibacterial activity. The Log Ratio Areas of *E. faecalis* and *S. aureus* are shown for monoculture experiments (black circles) and co-culture with β-lactamase deficient *E. coli* (red triangles), *E. coli* producing TEM-1 (green squares), and *E. coli* producing KPC-3 and TEM-1B enzymes (blue diamonds). A Hill-type model was not able to describe some of the data for *E. faecalis* AR Bank #0671 due to lack of bacterial killing.

The maximum β-lactam killing (E_max_) of *E. faecalis* and *S. aureus* is depicted in Figure 3. Against both *E. faecalis* isolates, ampicillin achieved an E_max_ ≥ 4.11 during monoculture experiments and during co-culture with *E. coli* that do not produce a β-lactamase. When *E. faecalis* AR Bank # 0573 and *E. faecalis* AR Bank # 0671 were separately cultured with TEM-1-producing *E. coli*, the E_max_ was reduced to 1.21 and 1.25, respectively. The E_max_ of ampicillin was 1.54 when *E. faecalis* AR Bank # 0573 was cultured with KPC-3/TEM-1B-producing *E. coli*, whereas no apparent killing was achieved against the second *E faecalis* isolate (E_max_ could not be defined). Against *S. aureus* ATCC 25923 alone, the E_max_ of cefazolin was 3.25, but the E_max_ declined down to 2.81 and 2.02 during co-culture with β-lactamase-deficient and β-lactamase-producing *E. coli*, respectively. The E_max_ of cefazolin ranged from 3.47–3.71 against *S. aureus* AR Bank # 0484 in each experiment with the exception that the E_max_ was reduced to 2.98 during co-culture with the KPC-3/TEM-1B-producing *E. coli* isolate.

#### Mechanism-based pharmacodynamic modelling of *S. aureus* and *E. coli* Co-culture

**FIGURE 4:**
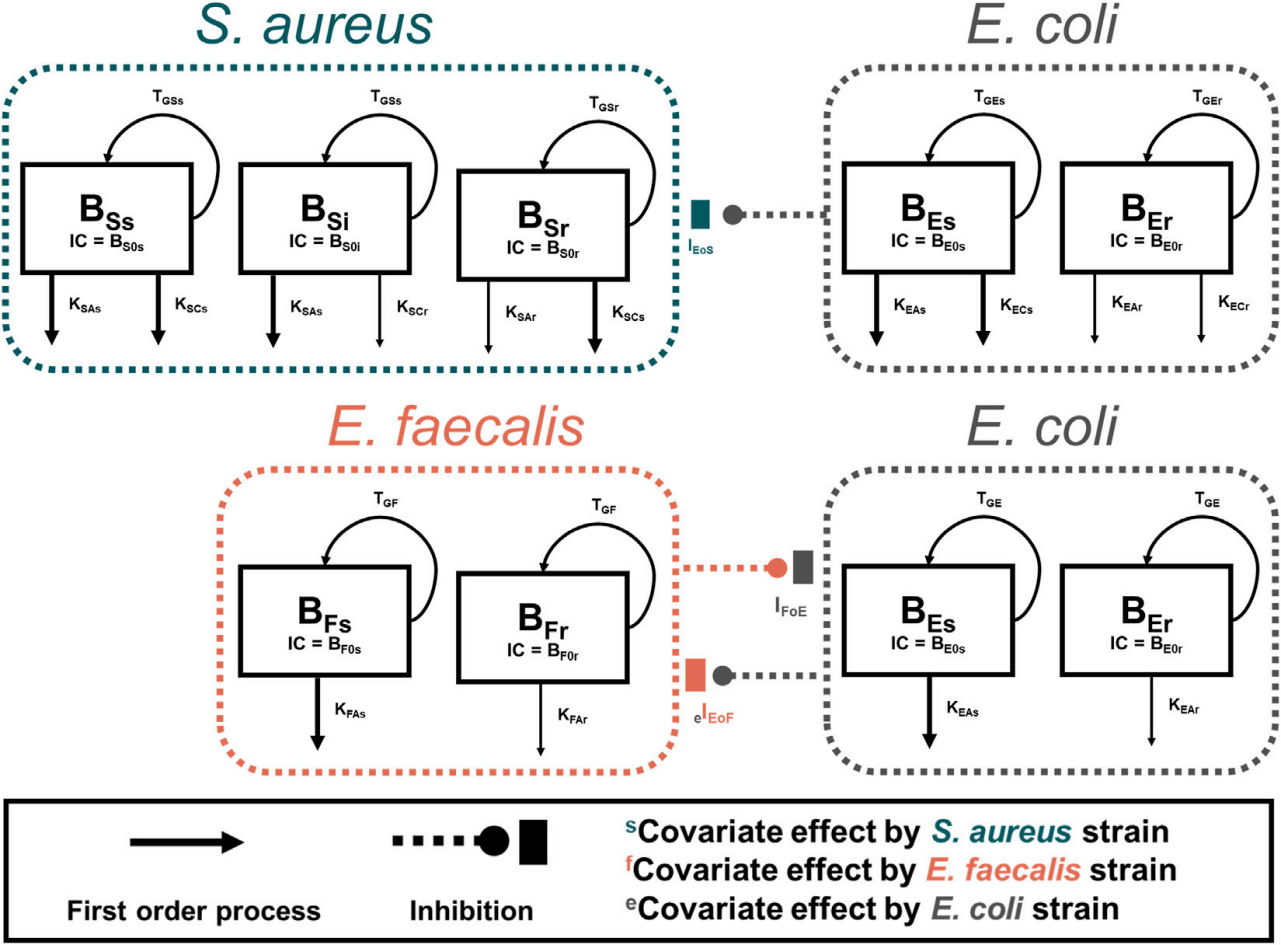
Model Diagrams. Two models were developed to describe each co-culture condition (*S. aureus*-*E. coli* and *E. faecalis-E. coli*). Experimental results of *S. aureus* in co-culture with *E. coli* were best described by using a subpopulation-based model where *S. aureus* was described by three sub-populations, principally differentiated by the sensitivity to either ampicillin or cefazolin. *E. faecalis* data were best characterized by two subpopulations with different rates of killing by ampicillin. For both models, *E. coli* total counts were best characterized by a two-subpopulation structure.

**FIGURE 5:**
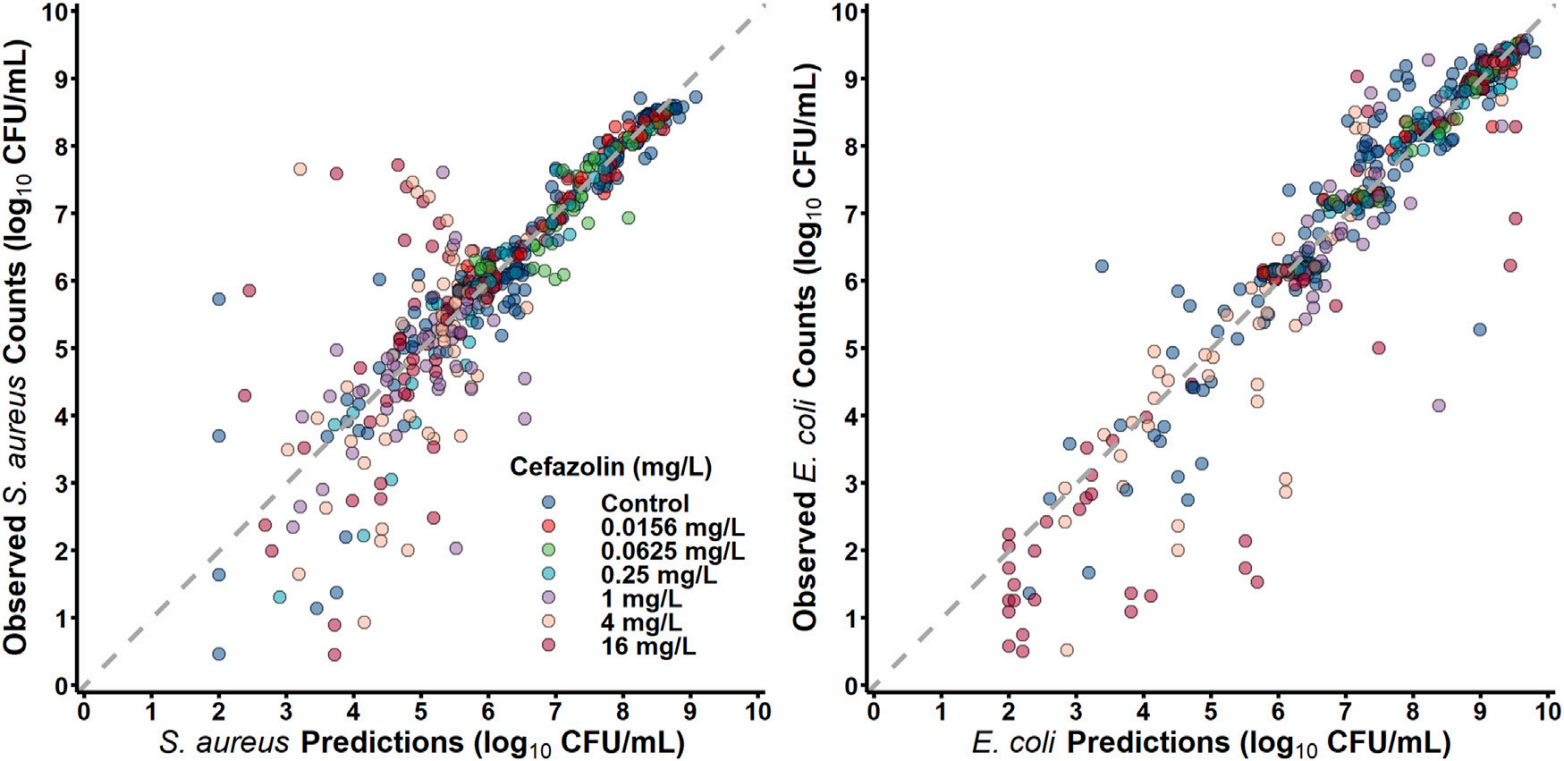
Observations versus individual predictions.

*S. aureus* in co-culture with *E. coli* was best described by a subpopulation-based model with susceptible and resistant sub-populations, which were characterized by unique mean generation times (MGT) and maximum rates of antibiotic-induced bacterial killing (K_max_) (Figure 4). Diagnostic plots showed that both *S. aureus* and *E. coli* total counts were well described by the model (Figure 5). Observed initial inocula for *S. aureus* and *E. coli* were 5.95 and 6.24 log_10_ CFU/mL with relative standard errors (RSEs) of 0.574 and 0.638, respectively, as compared to the target of six log_10_ CFU/mL (Table 1). For both *S. aureus* isolates, the “susceptible” subpopulations of the mechanism-based model had an estimated sensitivity (i.e., KC_50_) of 0.416 mg/L (15.9%) and 1.01 mg/L (36.6% RSE) for cefazolin and ampicillin, respectively. A strain-effect was observed with one isolate (*S. aureus* AR Bank # 0484), where the maximum rate of *S. aureus* killing by cefazolin was lowered by 0.357-fold (27.4%RSE) from 3.15 h^-1^ (22.7%RSE) to 1.12 h^-1^, as compared to *S. aureus* ATCC 25923. When in co-culture with *E. coli*, *S. aureus* experienced a reduction in its cefazolin K_max_ by 24.8% (23.1%RSE), indicating a reduction in drug effect.

**TABLE 1:** Model parameter estimates for *S. aureus-E. coli* Co-culture studies.

Parameter	Definition	Units	Estimate (%RSE)
$b_{mx,S}$	Maximum *S. aureus* population^a^	log_10_ CFU/mL	8.42 (1.05%)
$b_{S0}$	Initial *S. aureus* inoculum^b^	log_10_ CFU/mL	5.95 (0.574%)
$m_{Si}$	Mutation frequency for *S. aureus* intermediate subpopulation^c^		−3.32 (7.84%)
$m_{Sr}$	Mutation frequency for *S. aureus* resistant subpopulation^d^	-	−2.00 (12.6%)
$T_{GSs}$	Mean generation time of *S. aureus* susceptible/intermediate subpopulation^e^	min	69.6 (6.88%)
$T_{GSr}$	Mean generation time of *S. aureus* resistant subpopulation^f^	min	277 (18.4%)
$K_{mx,SCs}$	Max cefazolin killing rate, *S. aureus* susceptible/intermediate subpopulation	h^-1^	1.79 (7.66%)
$K_{mx,SCr}$	Max cefazolin killing rate, *S. aureus* resistant subpopulation	h^-1^	0.0610 (84.3%)
$C_{50,S}$	Cefazolin concentration for 50% *S. aureus* K_mx,SC_	mg/L	0.416 (15.9%)
$K_{mx,SAs}$	Max ampicillin killing rate*, S. aureus* susceptible/intermediate subpopulation	h^-1^	3.14 (22.7%)
$K_{mx,SAr}$	Max ampicillin killing rate, *S. aureus* resistant subpopulation	h^-1^	0.574 (32.9%)
$A_{50,S}$	Ampicillin concentration for 50% *S. aureus* K_mx,SA_	mg/L	1.01 (36.6%)
$b_{mx,E}$	Maximum *E. coli* population^g^	log_10_ CFU/mL	9.33 (0.354%)
$b_{E0}$	Initial *E. coli* inoculum^h^	log_10_ CFU/mL	6.24 (0.638%)
$m_{Er}$	Mutation Frequency for *E. coli* resistant subpopulation	-	−1.72 (4.37%)
$T_{GEs}$	Mean generation time of *E. coli* susceptible subpopulation^i^	min	102 (11.7%)
$T_{GEr}$	Mean generation time of *E. coli* resistant subpopulation^j^	min	29.3 (3.81%)
$\beta_{JL42}$	Covariate effect of *E. coli* JL42 (KPC-producer) on T_GEr_	-	−0.526 (9.63%)
$K_{\text{mx},EA}$	Max *E. coli* killing rate by ampicillin	h^-1^	2.14 (3.49%)
$A_{50,Es}$	Ampicillin concentration for 50% *E. coli* K_mx_, susceptible subpopulation	mg/L	7.32 (24.5%)
$A_{50,Er}$	Ampicillin concentration for 50% *E. coli* K_mx_, resistant subpopulation	mg/L	0.614 (32.4%)
$K_{mx,ECs}$	Max cefazolin killing rate, *E. coli* susceptible subpopulation	h^-1^	5.13 (15.1%)
$K_{mx,ECr}$	Max cefazolin killing rate, *E. coli* resistant subpopulation	h^-1^	0.901 (14.0%)
$C_{50,Es}$	Cefazolin concentration for 50% *E. coli* K_mx,ECs_	mg/L	7.02 (37.0%)
$C_{50,Er}$	Cefazolin concentration for 50% *E. coli* K_mx,ECr_	mg/L	5.11 (42.2%)
$I_{E}$	Reduction in *S.aureus* K_mx,SC_ by *E. coli*	-	0.248 (23.1%)
$b_{50,E}$	Log-transformed *E. coli* concentration for 50% I_E_	log10 CFU/mL	2 (fixed)
K_D_	Cell division rate constant	h_-1_	50 (fixed)
a_S_	Constant residual error of *S. aureus* observations	log10 CFU/mL	0.323 (4.81%)
a_E_	Constant residual error of *E. coli* observations	log10 CFU/mL	0.283 (4.96%)

^a^ IIV, estimated as 5.13 CV% (17.2%RSE)

^b^ IIV, estimated as 1.43 CV% (80.1%RSE)

^c^ IIV, estimated as 11.8 CV% (61%RSE)

^d^IIV, estimated as 15.0 CV% (82.6%RSE)

^e^IIV, estimated as 34.6 CV% (11.3%RSE)

^f^ IIV, estimated as 58.9 CV% (16.9%RSE)

g IIV, estimated as 1.10 CV% (46.5%RSE)

h IIV, estimated as2.96 CV% (20.9%RSE)

i IIV, estimated as 43.2 CV% (16.2%RSE)

j IIV, estimated as 8.49 CV% (20.3%RSE)

#### Pharmacodynamic modelling of *E. faecalis* and *E. coli* Co-culture

**FIGURE 6:**
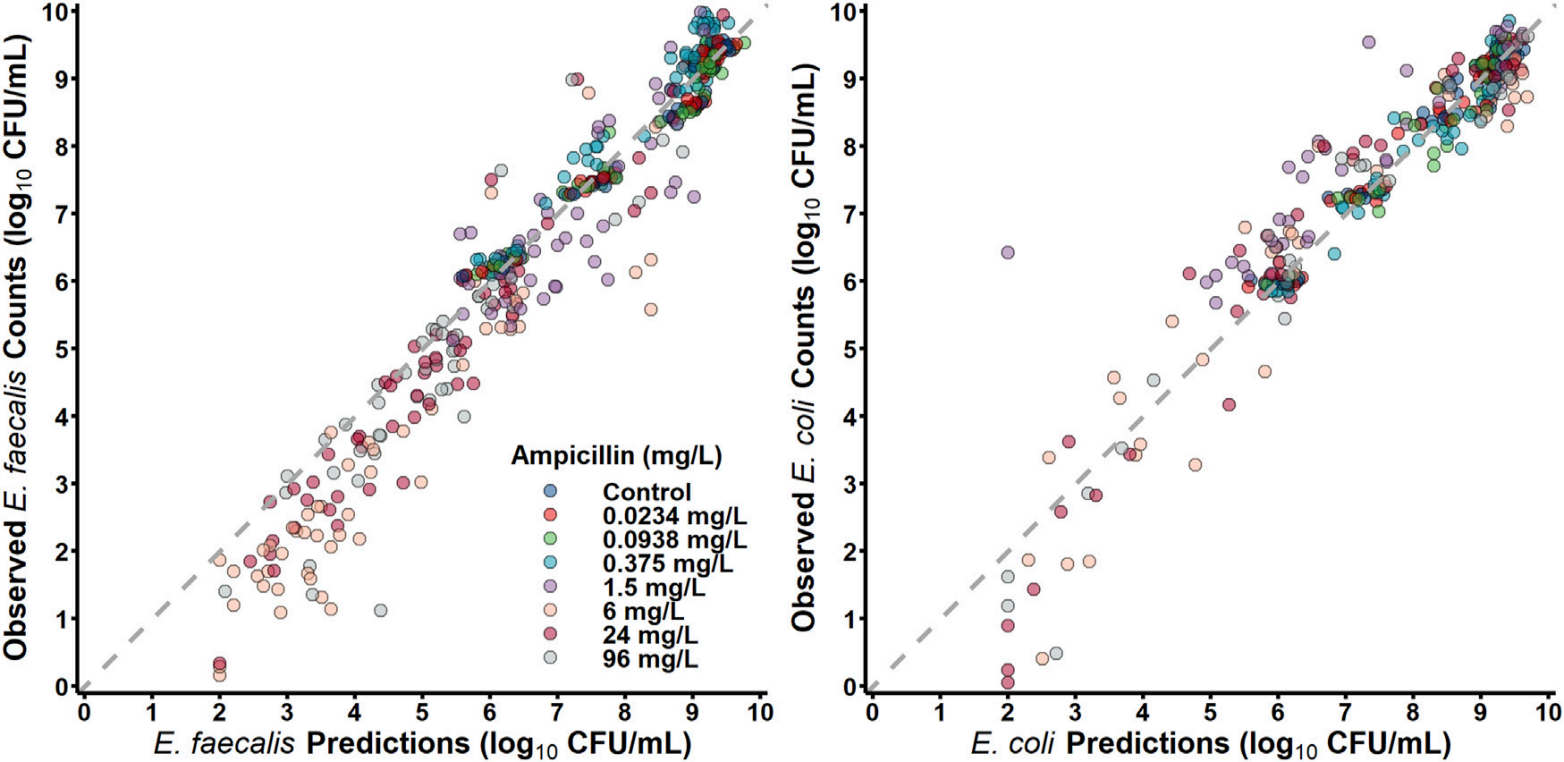
Observations *versus* individual predictions.

*E. faecalis* in co-culture with *E. coli* was best described with a subpopulation model that implemented susceptible- and resistant-subpopulations with unique maximum rates of ampicillin-induced killing (Figure 4). Diagnostics of the model predictions showed that both *E. faecalis* and *E. coli* data were well described (Figure 6). The observed starting inocula for *E. faecalis* and *E. coli* were 6.17 (0.472%RSE) and 6.03 (0.635%RSE) log_10_ CFU/mL, as compared to the target of six log_10_ CFU/mL (Table 2). β-lactamase-production by *E. coli* was found to reduce the maximum rate of ampicillin-induced killing of *E. faecalis* by 90.4% (0.978%RSE) *versus* a reduction of 70.6% (21.7%) for the β-lactamase-deficient *E. coli*. Maximum ampicillin-induced killing on *E. coli* (K_max_) was estimated to be 0.484 h^-1^, and was strongly influenced by isolate, with β-lactamase-deficient *E. coli* and TEM-1-producing *E. coli* having an estimated 8.33- and 2.75-fold increased rate of killing in comparison to the KPC-3/TEM-1B-producing *E. coli*, respectively.

**TABLE 2:** Model parameter estimates for *E. faecalis*-*E. coli* Co-culture studies.

Parameter	Definition	Units	Estimate (%RSE)
$b_{mx,F}$	Maximum *E. faecalis* population^a^	log_10_ CFU/mL	9.52 (0.625%)
$b_{F0}$	Initial *E. faecalis* inoculum^b^	log_10_ CFU/mL	6.17 (0.472%)
$m_{Fr}$	Mutation Frequency for *E. faecalis* resistant subpopulation	-	−3.74 (1.53%)
$T_{GF}$	Mean generation time of *E. faecalis*	min	39.9 (1.57%)
$K_{\text{mx},\text{FA}s}$	Max killing rate by ampicillin, *E. faecalis* susceptible subpopulation	h^-1^	2.70 (1.69%)
$K_{\text{mx},\text{FA}r}$	Max killing rate by ampicillin, *E. faecalis* resistant subpopulation	h^-1^	1.04 (1.91%)
$A_{50,F}$	Ampicillin concentration for 50% *E. faecalis* K_mx_	mg/L	4.70 (4.14%)
$I_{F}$	Reduction in *E. coli* K_mx,Es_ by *E. faecalis*	-	0.364 (5.13%)
$b_{50,F}$	Log-transformed *E. faecalis* concentration for 50% I_F_	log10 CFU/mL	2 (fixed)
H	Shape parameter for *E. faecalis* effect on *E. coli*	-	5 (fixed)
$b_{E0}$	Initial *E. coli* inoculum^c^	log_10_ CFU/mL	6.03 (0.635%)
$m_{Er}$	Mutation Frequency for *E. coli* resistant subpopulation	-	−6.50 (3.66%)
$T_{GE}$	Mean generation time of *E. coli*	min	38.0 (2.01%)
$K_{\text{mx},EAs}$	Max killing rate by ampicillin, *E. coli* susceptible subpopulation	h^-1^	0.484 (5.84%)
$\beta_{JL32}$	Covariate effect of JL32 on K_max,Es_	-	2.12 (2.52%)
$\beta_{JL33}$	Covariate effect of JL33 on K_max,Es_	-	1.01 (4.96%)
$K_{\text{mx},EAr}$	Max killing rate by ampicillin, *E. coli* resistant subpopulation	h^-1^	0.824 (3.97%)
$A_{50,E}$	Ampicillin concentration for 50% *E. coli* K_mx_	mg/L	0.961 (2.13%)
γ	Shape parameter for ampicillin killing of *E. coli*	-	4.87 (7.08%)
$I_{E}$	Reduction in *E. faecalis* K_mx,r_ by *E. coli*	%	0.904 (0.978%)
$\beta_{JL32}$	Covariate effect of JL32 on, I_E_ (on normal scale)	-	−0.938 (21.7%)
$b_{50,E}$	Log-transformed *E. faecalis* concentration for 50% I_E_	log10 CFU/mL	2 (fixed)
$H_{E}$	Shape parameter for *E. faecalis* effect on *E. coli*	-	5 (fixed)
K_D_	Cell division rate constant	h_-1_	50 (fixed)
a_F_	Constant residual error of *E. faecalis* observations	log10 CFU/mL	0.271 (3.84%)
a_E_	Constant residual error of *E. coli* observations	log10 CFU/mL	0.339 (4.65%)

^a^ IIV, fixed to 0.05.

^b^ IIV, estimated as 2.49% (15.2%RSE).

^c^ IIV, estimated as 2.05% (24.9% RSE)

## Discussion

Polymicrobial infections consisting of Enterobacterales and gram-positive pathogens are difficult to manage clinically, with optimal antibiotic strategies being unclear, especially in cases where one or more bacteria express β-lactamases or other antibiotic-modifying enzymes (Lenhard et al., 2019; Smith et al., 2021a). The situation is further complicated by the global spread of KPC-producing Enterobacterales, which are capable of inactivating the majority of β-lactam antibacterials (Hansen, 2021). Given the complexity of polymicrobial interactions, the present study sought to utilize novel mathematical modelling approaches to facilitate improved assessment of antibiotic action and potential methods to optimize therapy. The co-culture of *E. coli* with both *S. aureus* and *E. faecalis* resulted in significant protective effects that reduced β-lactam killing by 24.8%–90.4%. Furthermore, the extent of protection provided by *E. coli* was dependent on the type of β-lactamase harboured by the *E. coli*, with KPC-producing *E. coli* conferring the most substantial protection from β-lactams for two of the four gram-positive isolates. *After simultaneous fitting of all data, residual variability was estimated as <0.35 log*_*10*_ *CFU/mL for bacteria studied, which is a typical finding for time kill experiments.*

Although it is intuitive that the expression of β-lactamases may protect neighbouring pathogens from β-lactam exposure, the relationship between the production of drug-modifying enzymes and the impact on the pharmacodynamics of antibacterials against surrounding organisms is nuanced. A previous study observed that β-lactamase-producing *S. aureus* and *Branhamella catarrhalis* were capable of protecting *Streptococcus pneumoniae* from ampicillin *in vivo*, whereas *Haemophilus influenzae* did not augment the survival of the *S. pneumoniae* despite the production of β-lactamase enzymes (Renneberg and Walder, 1989). A subsequent *in vivo* investigation was able to confirm the inability of β-lactamase-producing *H. influenzae* to protect *S. pneumonia*e from an aminopenicillin (Westman et al., 2004). One variable that may impact the magnitude of a protective effect conferred by the production of drug-altering enzymes may be whether the β-lactamases are released into the extracellular space (Liao et al., 2014; Liao et al., 2015). In a prior *in vitro* investigation, aminoglycoside modifying enzyme-producing *E. faecalis* was not capable of appreciably protecting neighbouring gram-negative pathogens from gentamicin despite exposure of *E. faecalis* to lethal concentrations of ampicillin in an attempt to liberate intracellular enzymes (McMurtry et al., 2021). The relationship between resistance mechanisms and the pharmacodynamics of antibacterials during polymicrobial infections is therefore complex, and investigations of specific pathogen relationships and resistance mechanisms are likely needed to optimize antibacterial selection during polymicrobial infections. In the current study, not only were β-lactamase-producing *E. coli* capable of protecting *S. aureus* and *E. faecalis* from β-lactams, but penicillin-resistant *S. aureus* also demonstrated the ability to protect β-lactamase-deficient *E. coli* from drug exposure as well.

The management of polymicrobial infections has become further complicated by the increased prevalence of carbapenem resistance among drug-resistant gram-negative pathogens. Carbapenem-resistant nonfermenting organisms have already demonstrated the ability to protect neighboring pathogens from β-lactam exposure. *A. baumannii*, for example, shielded *E. coli* and *S. aureus* from carbapenems in two separate investigations (Liao et al., 2014; Smith et al., 2021b). Similarly, *Stenotrophomonas maltophilia* protected *Serratia marcscens* from imipenem and ceftazidime using inducible β-lactamase production (Kataoka et al., 2003). An equally frightening scenario, however, is the involvement of CRE in a polymicrobial infection. The transmissible spread of carbapenem resistance among Enterobacterales strains internationally has generated considerable concern for the global healthcare system, with KPC-producing Enterobacterales now representing some of the most relevant CRE internationally (Anonymous. WHO, 2017; Hansen, 2021; Centers for Disease Control, 2023). In the current investigation, the KPC-producing *E. coli* generated the most pronounced protective effect for both *S. aureus* and *E. faecalis*, indicating that CRE are capable of shielding neighboring pathogens from β-lactam exposure in a manner analogous to nonfermenting pathogens.

In the present study, the variability of the β-lactam protective effect conferred by the different *E. coli* isolates suggests that the management of polymicrobial infections may potentially be improved if therapy can be individualized for a specific polymicrobial community. Clinicians are beginning to recognize that polymicrobial communities have likely been underdiagnosed in the past, and new molecular techniques may allow for more rapid identification of a polymicrobial infectious process (Athamanolap et al., 2018; Zhang et al., 2019). An Israeli study found that about one in three cases of urosepsis at the authors’ institution were polymicrobial, with polymicrobial urosepsis being associated with a higher mortality rate than monomicrobial infections (28% *versus* 15%, *p* < 0.05) (Siegman-Igra et al., 1994). The most common gram-negative pathogens in polymicrobial urosepsis were Enterobacterales, whereas *Enterococcus* species were the most common gram-positive organisms. Similarly, another group observed that neonatal patients with polymicrobial bacteremia experienced a higher mortality rate in comparison to monomicrobial bacteremia (adjusted odds ratio 4.3, 95% CI 1.8–10.2) (Pammi et al., 2014). *Enterococcus* species and *Klebsiella* species were the most commonly encountered pathogens in polymicrobial bacteremia, and both organisms were isolated from polymicrobial infections over twice as frequently than from monomicrobial infections. Further translational and clinical investigations into the optimal antibacterial selection and dosing during polymicrobial infections may allow for clinical practice guidelines that address situations such as polymicrobial urinary tract infections, bacteremia, and other sites of infection that are traditionally viewed as monomicrobial.

The current study has multiple limitations that should be considered when interpreting the results of the investigation. Firstly, all of the experiments were completed *in vitro* using static concentrations of β-lactam antibacterials. Secondly, a limited number of isolates that were selected based on their production of β-lactamase enzymes were evaluated in the study. Lastly, there are many β-lactamase enzymes produced by Enterobacterales that were not included in the investigation. Future *in vivo* studies that utilize a diverse collection of pathogens isolated from polymicrobial infections will likely be able to expand upon the results of the present investigation.

In closing, the current investigation affirms the ability of β-lactamase-producing Enterobacterales to protect neighbouring gram-positive pathogens from β-lactam exposure. The degree of such a shielding effect likely depends on the enzymes produced by a given isolate, with KPC enzymes demonstrating a marked ability to augment the survival of adjacent organisms. Penicillin-resistant *S. aureus* also demonstrated the ability to protect β-lactamase-deficient *E. coli* from ampicillin exposure, highlighting the complexity of polymicrobial interactions. Further investigations that evaluate the optimal antibacterial regimens to use against specific groups of pathogens may assist with clinical decision making during polymicrobial infections.

## Materials and methods

### Bacterial isolates

Three *E. coli* isolates, two *S. aureus* isolates, and 2 *E. faecalis* isolates were included in the investigation. Each *E. coli* isolate possessed a different β-lactamase status, such that one organism did not produce a β-lactamase (*E. coli* AR Bank #0017, MIC_ampicillin_ < 1 mg/L and MIC_cefazolin_ = 2 mg/L), one isolate produced TEM-1 (*E. coli* AR Bank #0019, MIC_ampicillin_ > 32 mg/L and MIC_cefazolin_ = 8 mg/L), and the final isolate produced KPC-3 and TEM-1B (*E. coli* AR Bank #0114, MIC_ampicillin_ > 32 mg/L and MIC_cefazolin_ > 32 mg/L). Both *S. aureus* isolates were susceptible to cefazolin (ATCC 25923 and *S. aureus* AR Bank #0484) and both *E. faecalis* isolates were susceptible to ampicillin (*E. faecalis* AR Bank #0573 and *E. faecalis* AR Bank #0671).

### Time-killing experiments

**FIGURE 7:**
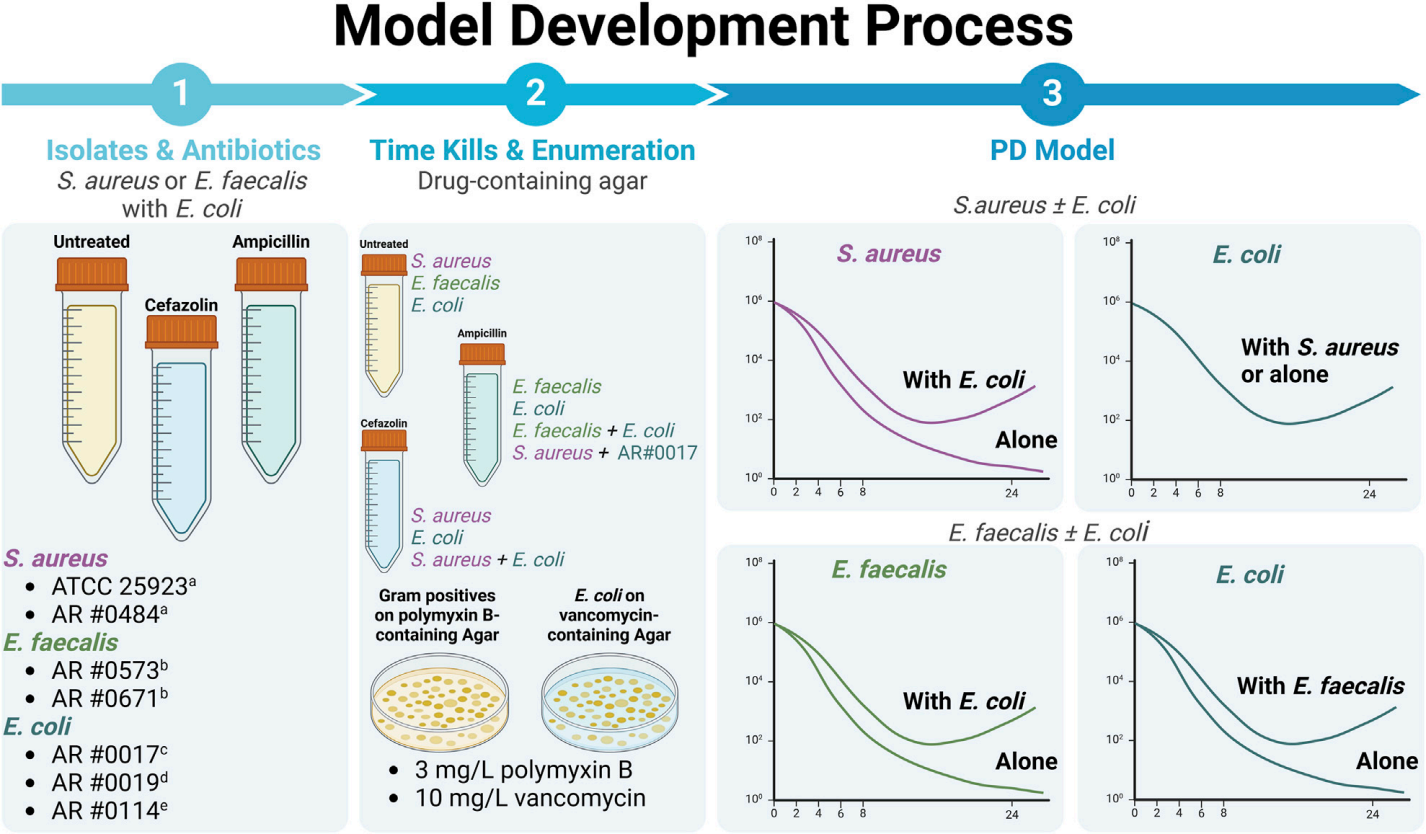
Our overall methodology involved detailed study of two different gram-positive bacteria, *S. aureus* and *E. faecalis*, alone and in co-culture with three different *E. coli* expressing different beta-lactamases. All experiments were performed using all possible combinations of *S. aureus* and *E. coli* strains along with all possible combinations of *E. faecalis* and *E. coli* strains, unless otherwise noted. Drug exposure were based on the typical first-line agent for each gram-positive. (i.e., primarily focused on use of cefazolin against *S. aureus* and ampicillin against *E. faecalis*). Please note that graphs of different bacterial killing effects are representative, only. ^a^Cefazolin susceptible. ^b^Ampicillin susceptible. ^c^Non-beta-lactamase producer. ^d^TEM-1-producing. ^e^KPC-3- and TEM-1B-co-producing (Created with BioRender.com).

Time-killing experiments were conducted over 24 h as described previously (Smith et al., 2021b). Generally, isolates were originally studied in mono-culture, then, to evaluate possible bacteria-bacteria interactions, studied in combination as either *S. aureus*-*E. coli* or *E. faecalis*-*E. coli* combinations (Figure 7). In brief, overnight cultures were used to create a ∼10^6^ CFU/mL inoculum of bacteria suspended in cation-adjusted Brain Heart Infusion broth. Each *S. aureus* and *E. faecalis* isolate was evaluated alone and during co-culture experiments in which the gram-positive organism was grown with one of the 3 *E. coli* isolates. In co-culture experiments, a 1:1 ratio of two organisms was used such that a total inoculum of 2 × 10^6^ CFU/mL was achieved consisting of 1 × 10^6^ CFU/mL of each pathogen. A concentration array of cefazolin ranging from 0.016–16 mg/L was used for experiments involving *S. aureus*, whereas ampicillin concentrations ranging from 0.023 to 96 mg/L were used for *E. faecalis* experiments. The cefazolin and ampicillin drug arrays were also evaluated against each *E. coli* isolate grown alone. A total suspension of 20 mL was contained in 50 mL conical tubes that were incubated at 37°C with constant shaking. At 0, 2, 4, 6, 8, and 24 h, 100 mcl samples were collected from the conical tubes, serially diluted in saline, and plated onto Brain Heart Infusion agar imbued with 8 mg/L of polymyxin B. During co-culture experiments, diluted samples were also plated onto Mueller-Hinton Agar impregnated with 4 mg/L of vancomycin to quantify the amount of *E. coli*. Plates were incubated at 37°C for 24 h and then used for viable cell counting.

### Empiric pharmacokinetics/pharmacodynamics analysis

In order to integrate the data obtained from time-killing experiments into a single quantifiable analysis, a mathematical approach was used to calculate maximum antibacterial activity during monoculture and co-culture conditions as described previously (McMurtry et al., 2021). In brief, the area under the CFU curve was calculated for each ampicillin or cefazolin concentration in each time-killing experiment using Systat Software version 14.0. The Log Ratio Area was then calculated by normalizing the area under the CFU curve of each drug concentration using the corresponding growth control (Eq. 1). Lastly, a Hill-type mathematical model was then used to describe the data, where E_max_ represents the maximum killing of either ampicillin or cefazolin (Eq. 2).

$$\mathrm{Log}\text{ Ratio Area}={\;\log}_{10}\left(\frac{{\text{AUCFU}}_{\text{drug}}}{A{\text{UCFU}}_{\text{growth control}}}\right)\geq$$ (1)

$$E={\;E}_{0}\;‐\;\frac{E_{\max}\;•\;{\left(C\right)}^{H}}{{\left({\text{EC}}_{50}\right)}^{H}+{\left(C\right)}^{H}}$$ (2)

### Pharmacokinetics/pharmacodynamics modelling

Data were modelled using Monolix (2022R1, Lixoft, Antony, France) by the stochastic approximation expectation maximization algorithm. Standard errors and likelihood were calculated using the linearization method, and observations below the limit of quantification (<10^2^ CFU/mL) were modelled as censored data. In general, the model development process for the subpopulation models was performed stepwise. First, resistant subpopulations were characterized by a mutation frequency parameter which identifies the starting concentration of the resistant bacterial subpopulation. Resistant subpopulations were then tested for differences in maximum killing rate (k_max_), sensitivity to the antibiotic of interest (KC_50_), or both, as done previously. After identifying parameters of antibiotic action, we tested for differences in growth rate between the subpopulations. To ensure the most parsimonious model is selected, likelihood ratio testing (for nested models) or comparison of the Schwartz Criterion was used. The mechanism-based model for co-culture conditions were performed as described above by first developing a base model for each bacterium in monoculture assuming a susceptible and resistant subpopulation with identical growth rates. Hill-type functions were utilized to describe β-lactam killing effects, based on previous studies that demonstrated saturable killing and underlying mechanism of (Regoes et al., 2004). Unique mechanistic interactions between bacteria were tested as either unidirectional or bidirectional effects on either the bacterial growth rate or antibiotic killing rate. Interactions were included based on reduction in model AIC. Given known differences in β-lactamase production, enzyme status was tested for statistical significance as a pharmacodynamic covariate on drug sensitivity (i.e., KC_50_) or maximum effect (i.e., K_max_). *Because most experiments were performed in singlicate, we characterized experimental variability using constant, residual variability parameters that are informed by all datapoints collected.*

## Data Availability

The raw data supporting the conclusion of this article will be made available by the authors, without undue reservation.
