# Supplementary material for: Influence of β-lactam pharmacodynamics on the systems microbiology of gram-positive and gram-negative polymicrobial communities
Source: Front Pharmacol. 2024 Jun 4;15:1339858. doi: 10.3389/fphar.2024.1339858 (PMC11183306; doi:10.3389/fphar.2024.1339858)

**SUPPLEMENTAL MATERIALS**

**Influence of β-lactam Pharmacodynamics on the Systems Microbiology of Gram-Positive and Gram-Negative Polymicrobial Communities**

Nicholas M. Smith^1^, Harpreet Kaur^2^, Ravneet Kaur^2^, Trisha Minoza^2^, Michael Kent^2^, Ayeh Barekat^2^, Justin R. Lenhard^2,*^

^1^School of Pharmacy and Pharmaceutical Sciences, University at Buffalo, NY, USA

^2^California Northstate University College of Pharmacy, Elk Grove, CA, USA^c^

*Correspondence:

Justin R. Lenhard

California Northstate University College of Pharmacy

Elk Grove, CA, USA

Phone: (916) 686 – 8007, Email: [Justin.Lenhard@cnsu.edu](mailto:Justin.Lenhard@cnsu.edu)

**Keywords**: polymicrobial infections; Staphylococcus aureus; Enterococcus faecalis; Escherichia coli; Systems Microbiology; mathematical modeling; pharmacodynamics; beta-lactams

**Equations: *S. aureus* + *E. coli Co-culture studies.***

*Global Transformation of Variables*

| *S. aureus* |  |
| --- | --- |
| $K_{\text{GSs}}=\frac{60}{T_{GSs}}$ | **1** |
| $K_{\text{GSr}}=\frac{60}{T_{GSr}}$ | **2** |
| $B_{mx,S}={10}^{b_{mx,S}}$ | **3** |
| $B_{S0}={10}^{b_{s0}}$ | **4** |
| $M_{si}={10}^{m_{si}}$ | **5** |
| $M_{sr}={10}^{m_{sr}}$ | **6** |
| $B_{S0i}=B_{S0}\cdot M_{si}$ | **7** |
| $B_{S0r}=B_{S0}\cdot M_{sr}$ | **8** |
| $B_{S0s}=B_{S0}-B_{S0r}-B_{S0i}$ | **9** |
| *E. coli* |  |
| $K_{\text{GEs}}=\frac{60}{T_{GEs}}$ | **10** |
| $K_{\text{GEr}}=\frac{60}{T_{GEr}}$ | **11** |
| $B_{mx,E}={10}^{b_{mx,E}}$ | **12** |
| $B_{E0}={10}^{b_{E0}}$ | **13** |
| $M_{Er}={10}^{m_{Er}}$ | **14** |
| $B_{E0r}=B_{E0}\cdot M_{Er}$ | **15** |
| $B_{E0s}=B_{E0}-B_{E0r}$ | **16** |
| $B_{\text{50,E}}={10}^{b_{50,E}}$ | **17** |

*Equations Governing Drug Effect*

| $K_{SAs}=\frac{K_{mx,SAs}\cdot A}{A_{\text{50,S}}+A}$ | **18** |
| --- | --- |
| $K_{SAr}=\frac{K_{mx,SAr}\cdot A}{A_{\text{50,S}}+A}$ | **19** |
| $I_{EoS}=\frac{I_{\text{E}}\cdot B_{\text{etot}}}{B_{50,E}+B_{\text{etot}}}$ | **20** |
| $K_{SCs}=\frac{K_{mx,SCs}\cdot C}{C_{\text{50,S}}+C}\cdot I_{EoS}$ | **21** |
| $K_{SCr}=\frac{K_{\text{mx,SCr}}\cdot C}{C_{\text{50,S}}+C}\cdot I_{EoS}$ | **22** |
| $K_{EAs}=\frac{K_{\text{mx,EA}}\cdot A}{A_{\text{50,Es}}+A}$ | **23** |
| $K_{EAr}=\frac{K_{\text{mx,EA}}\cdot A}{A_{\text{50,Er}}+A}$ | **24** |
| $K_{ECs}=\frac{K_{\text{mx,EC}}\cdot C}{C_{\text{50,Es}}+C}$ | **25** |
| $K_{ECr}=\frac{K_{\text{mx,EC}}\cdot C}{C_{50,Er}+C}$ | **26** |

*Equations Governing Bacterial Growth*

| $B_{stot}=B_{ss1}+B_{ss2}+B_{si1}+B_{si2}+B_{sr1}+B_{sr2}$ | **27** |
| --- | --- |
| $B_{etot}=B_{es1}+B_{es2}+B_{er1}+B_{er2}$ | **28** |
| $F_{\text{rs}}=2*\left( 1-\frac{B_{stot}}{B_{mx,S}+B_{stot}} \right)$ | **29** |
| $F_{\text{re}}=2*\left( 1-\frac{B_{etot}}{B_{mx,E}+B_{etot}} \right)$ | **30** |

*Differential Equations*

| $\frac{dB_{ss1}}{dt}=F_{rs}*K_{D}*B_{ss2}-K_{\text{GSs}}*B_{ss1}-\left( K_{SAs}+K_{SCs} \right)*B_{ss1}$  $B_{ss1}\left( 0 \right)=B_{ss,0}$ | **31** |
| --- | --- |
| $\frac{dB_{ss2}}{dt}=-K_{D}\cdot B_{ss2}+K_{\text{GSs}}\cdot B_{ss1}-\left( K_{SAs}+K_{SCs} \right)\cdot B_{ss2}$  $B_{ss2}\left( 0 \right)=0$ | **32** |
| $\frac{dB_{si1}}{dt}=F_{rs}*K_{D}*B_{si2}-K_{\text{GSs}}*B_{si1}-\left( K_{SAs}+K_{SCr} \right)*B_{si1}$  $B_{si1}\left( 0 \right)=B_{si,0}$ | **33** |
| $\frac{dB_{si2}}{dt}=-K_{D}\cdot B_{si2}+K_{\text{GSs}}\cdot B_{si1}-\left( K_{SAs}+K_{SCr} \right)\cdot B_{si2}$  $B_{sr2}\left( 0 \right)=0$ | **34** |
| $\frac{dB_{sr1}}{dt}=F_{rs}*K_{D}*B_{sr2}-K_{\text{GSr}}*B_{sr1}-\left( K_{SAr}+K_{SCs} \right)*B_{sr1}$  $B_{sr1}\left( 0 \right)=B_{sr,0}$ | **35** |
| $\frac{dB_{sr2}}{dt}=-K_{D}\cdot B_{sr2}+K_{\text{GSr}}\cdot B_{sr1}-\left( K_{SAr}+K_{SCs} \right)\cdot B_{sr2}$  $B_{sr2}\left( 0 \right)=0$ | **36** |
| $\frac{dB_{es1}}{dt}=F_{re}*K_{D}*B_{es2}-K_{\text{GEs}}*B_{es1}-\left( K_{EAs}+K_{ECs} \right)*B_{es1}$  $B_{si1}\left( 0 \right)=B_{es,0}$ | **37** |
| $\frac{dB_{es2}}{dt}=-K_{D}\cdot B_{es2}+K_{\text{GEs}}\cdot B_{es1}-\left( K_{EAs}+K_{ECs} \right)\cdot B_{es2}$  $B_{sr2}\left( 0 \right)=0$ | **38** |
| $\frac{dB_{er1}}{dt}=F_{re}*K_{D}*B_{er2}-K_{\text{GEr}}*B_{er1}-\left( K_{EAr}+K_{ECr} \right)*B_{er1}$  $B_{er1}\left( 0 \right)=B_{er,0}$ | **39** |
| $\frac{dB_{er2}}{dt}=-K_{D}\cdot B_{er2}+K_{\text{GEr}}\cdot B_{er1}-\left( K_{EAr}+K_{ECr} \right)\cdot B_{er2}$  $B_{er2}\left( 0 \right)=0$ | **40** |

**Equations: *E. faecalis* + *E. coli Co-culture studies.***

*Global Transformation of Variables*

| *E. faecalis* |  |
| --- | --- |
| $K_{\text{GF}}=\frac{60}{T_{GF}}$ | **41** |
| $B_{mx,F}={10}^{b_{mx,F}}$ | **42** |
| $B_{F0}={10}^{b_{F0}}$ | **43** |
| $M_{Fr}={10}^{m_{Fr}}$ | **44** |
| $B_{F0r}=B_{F0}\cdot M_{Fr}$ | **45** |
| $B_{F0s}=B_{S0}-B_{S0r}$ | **46** |
| $B_{50,F}={10}^{LGB_{50,F}}$ | **47** |
| E. coli |  |
| $K_{\text{GEs}}=\frac{60}{T_{GEs}}$ | **48** |
| $B_{mx,E}={10}^{b_{mx,E}}$ | **49** |
| $B_{E0}={10}^{b_{E0}}$ | **50** |
| $M_{Er}={10}^{m_{Er}}$ | **51** |
| $B_{E0r}=B_{E0}\cdot M_{Er}$ | **52** |
| $B_{E0s}=B_{E0}-B_{E0r}$ | **53** |
| $B_{\text{50,E}}={10}^{LGB_{\text{50,E}}}$ | **54** |

*Equations Governing Drug Effect*

| $I_{FoE}=\frac{I_{F}\cdot B_{\text{tot,F}}^{H}}{B_{50,F}^{H}+B_{\text{tot,F}}^{H}}$ | **55** |
| --- | --- |
| $I_{EoF}=\frac{I_{\text{E}}\cdot B_{\text{tot,E}}}{B_{50,E}+B_{\text{tot,E}}}$ | **56** |
| $K_{FS}=\frac{K_{\text{mx,FAs}}\cdot A}{A_{\text{50,F}}+A}$ | **57** |
| $K_{FR}=\frac{K_{\text{mx,FAr}}\cdot A}{A_{\text{50,F}}+A}\cdot(1-I_{EoF})$ | **58** |
| $K_{ES}=\frac{K_{\text{mx,EAs}}\cdot A^{\gamma}}{A_{\text{50,E}}^{\gamma}+A^{\gamma}}\cdot(1-I_{FoE})$ | **59** |
| $K_{ER}=\frac{K_{\text{mx,Er}}\cdot A^{\gamma}}{A_{\text{50,E}}^{\gamma}+A^{\gamma}}$ | **60** |

*Equations Governing Bacterial Growth*

| $B_{tot,F}=B_{Fs1}+B_{Fs2}+B_{Fr1}+B_{Fr2}$ | **61** |
| --- | --- |
| $B_{tot,E}=B_{es1}+B_{es2}+B_{er1}+B_{er2}$ | **62** |
| $F_{\text{r}F}=2\cdot\left( 1-\frac{B_{tot,F}}{B_{mx,F}+B_{tot,F}} \right)$ | **63** |
| $F_{\text{rE}}=2\cdot\left( 1-\frac{B_{tot,E}}{B_{mx,\text{E}}+B_{tot,E}} \right)$ | **64** |

*Differential Equations*

| $\frac{dB_{Fs1}}{dt}=F_{rF}\cdot K_{D}\cdot B_{Fs2}-K_{F}*B_{ss1}-K_{Fs}\cdot B_{Fs1}$  $B_{Fs1}\left( 0 \right)=B_{Fs,0}$ | **65** |
| --- | --- |
| $\frac{dB_{Fs2}}{dt}=-K_{D}\cdot B_{Fs2}+K_{\text{F}}\cdot B_{Fs1}-K_{Fs}\cdot B_{Fs2}$  $B_{Fs2}\left( 0 \right)=0$ | **66** |
| $\frac{dB_{Fr1}}{dt}=F_{rF}\cdot K_{D}\cdot B_{Fr2}-K_{\text{F}}\cdot B_{Fr1}-K_{Fr}\cdot B_{Fr1}$  $B_{Fr1}\left( 0 \right)=B_{Fr,0}$ | **67** |
| $\frac{dB_{Fr2}}{dt}=-K_{D}\cdot B_{Fr2}+K_{F}\cdot B_{Fr1}-K_{Fr}\cdot B_{Fr2}$  $B_{Fr2}\left( 0 \right)=0$ | **68** |
| $\frac{dB_{Es1}}{dt}=F_{rE}\cdot K_{D}\cdot B_{Es2}-K_{E}\cdot B_{Es1}-K_{Es}\cdot B_{Es1}$  $B_{si1}\left( 0 \right)=B_{es,0}$ | **69** |
| $\frac{dB_{Es2}}{dt}=-K_{D}\cdot B_{Es2}+K_{E}\cdot B_{Es1}-K_{Es}\cdot B_{Es2}$  $B_{Es2}\left( 0 \right)=0$ | **70** |
| $\frac{dB_{Er1}}{dt}=F_{re}\cdot K_{D}\cdot B_{Er2}-K_{E}\cdot B_{Er1}-K_{Er}\cdot B_{Er1}$  $B_{er1}\left( 0 \right)=B_{er,0}$ | **71** |
| $\frac{dB_{Er2}}{dt}=-K_{D}\cdot B_{Er2}+K_{E}\cdot B_{er1}-K_{Er}\cdot B_{Er2}$  $B_{er2}\left( 0 \right)=0$ | **72** |

Figure S1: Time-killing plots depicting the activity of ampicillin against β-lactamase-deficient *E. coli* cultured alone or with *E. faecalis.* The quantity of *E. coli* is depicted for both monoculture (solid lines) and co-culture (dashed line) experiments


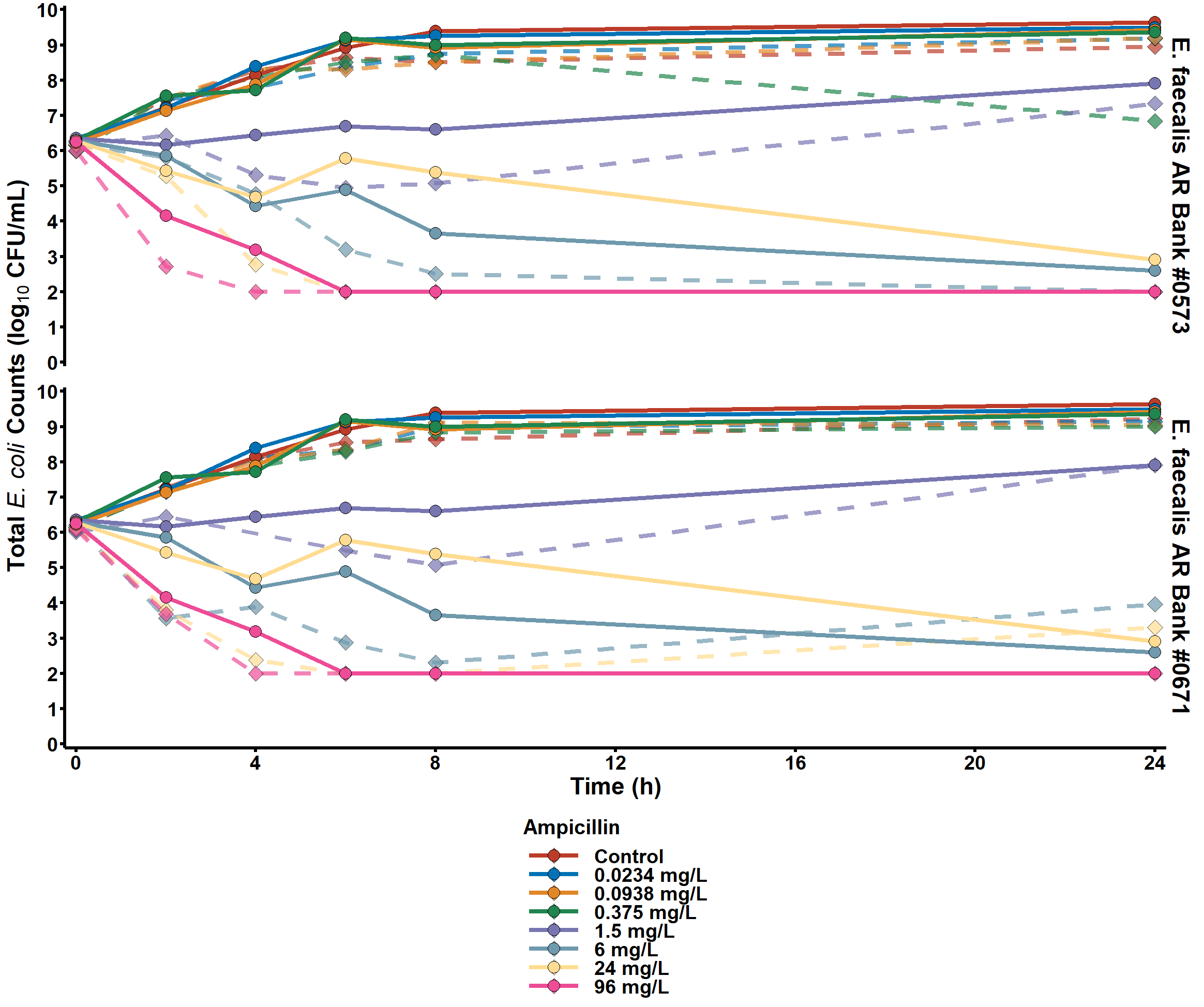


Figure S2: Time-kill plots depicting the activity of ampicillin against β-lactamase-deficient *E. coli* cultured alone or with *S. aureus*. The quantity of *E.coli* is displayed for monoculture (solid lines) or co-culture (dashed line) experiments. *S. aureus* ATCC 25923 was susceptible to penicillins, whereas *S. aureus* AR Bank # 0484 was penicillin-resistant.


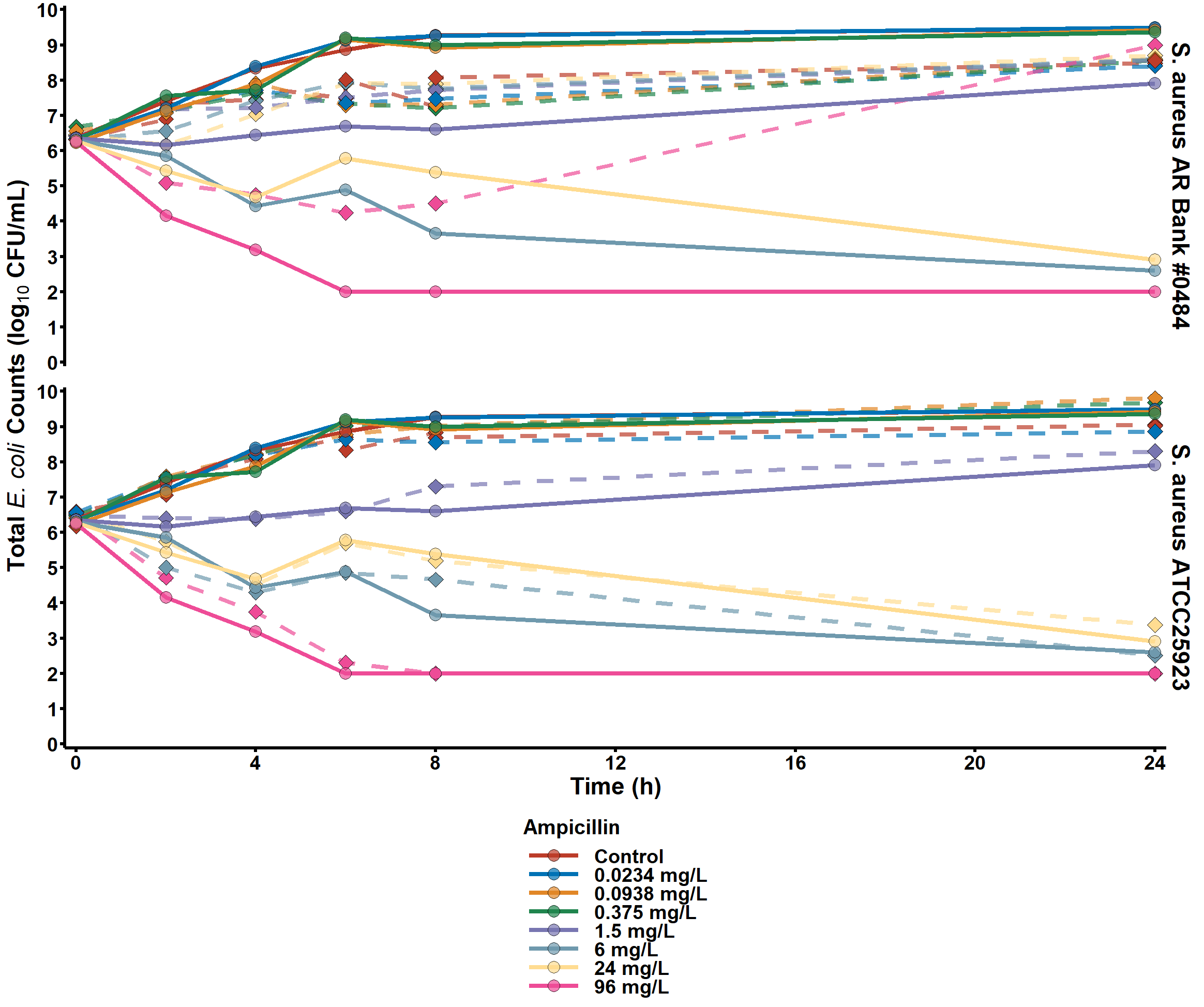


Figure S3: Time-killing plots depicting the activity of cefazolin against β-lactamase-deficient *E. coli* cultured alone or with *S. aureus.* The quantity of *E. coli* is depicted for both monoculture (solid lines) and co-culture (dashed line) experiments.
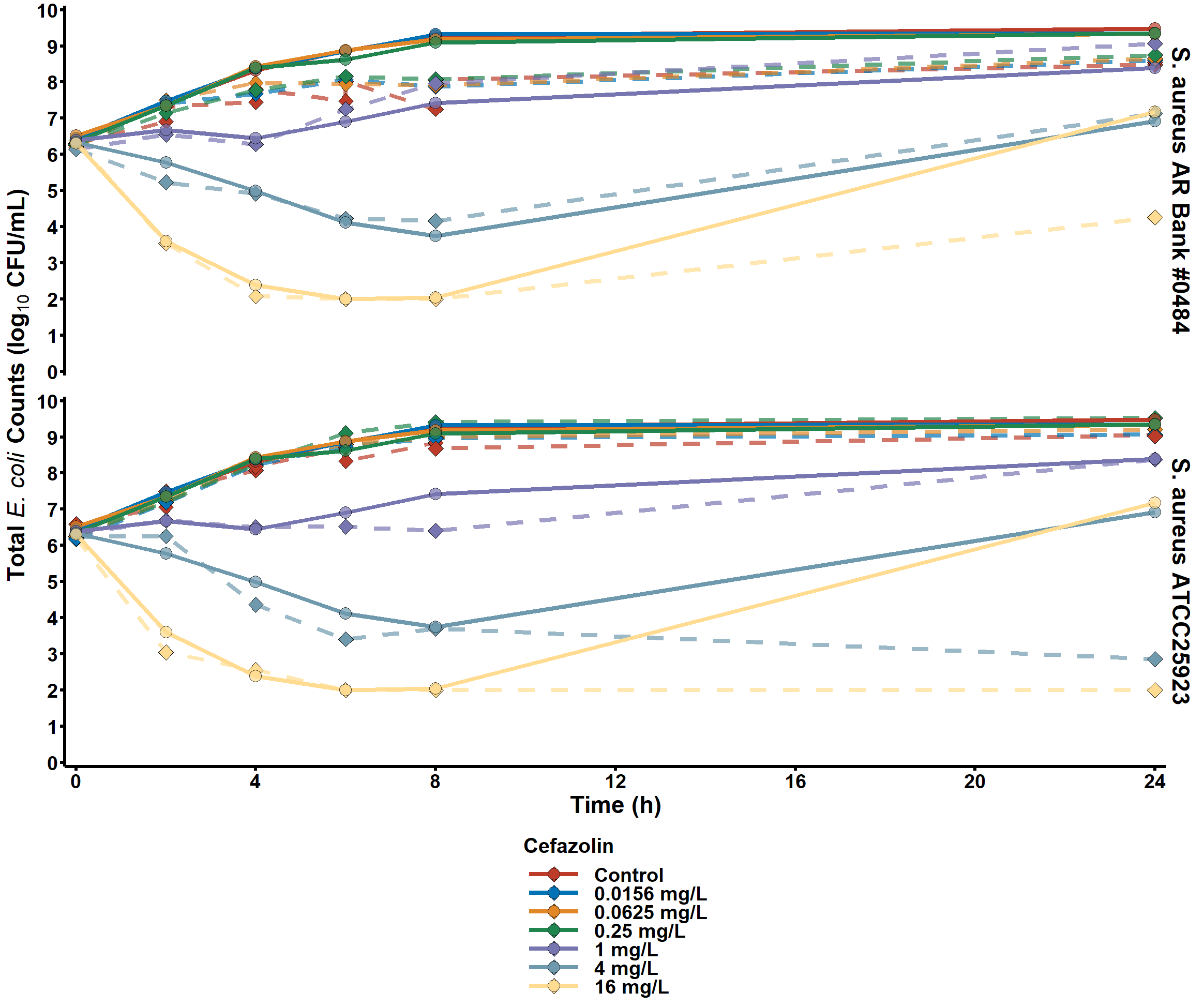

Supplement: Supplementary file 1 [file DataSheet1.docx]
